# An Act to Incorporate Medical Societies for the Purpose of Regulating the Practice of Physic and Surgery in Ohio

**Published:** 1829

**Authors:** 


Art. XI.—*An Act to incorporate Medical Societies for the purpose of regulating the Practice of Physic and Surgery in Ohio. Together with the proceedings of the General Medical Society. Columbus:* 1829.

In the different states of our *imperium in imperio*, a very different policy prevails, in relation to the medical profession. In some of them, as our neighbour, Kentucky, no legislative enactments on the subject have yet appeared; while in others it has been a point of policy to unite the whole body of medical practitioners, into one corporation. This is the case in Ohio, where successive laws for this purpose have been enacted, during a period of nearly 20 years. Unfortunately for the object in view, these laws have superseded each other, before any one of them, had been in operation for a sufficient length of time, to afford the means of judging of its excellence or its defects. The present act has, however, been suffered to remain since 1824.

By this statute the state is divided into twenty odd medical districts, the physicians in each of which, constitute an independent corporation or society. By this act it was ordered, that each district society, should in 1827, elect one delegate, to a convention, to be holden at Columbus, in January 1828; which convention should have power to form a general or state medical society, to be composed of a member from each district. The convention met, and its principal act was the formation of a constitution for such a society; which was ordered to assemble at Columbus, on the 5th of Janury 1829. The meeting accordingly took place:—present, Drs. Bush, Dunlavy, Martin, Coleman, Smith, Manning, Pinkerton, Parsons, Cotton, M'Neill, Flanner, Dickson, Bissel, Wright, Hawley, Brice, and Thompson.

By the statute of the state, this confederate body, denominated the 'General Medical Society of the State of Ohio,' is a body corporate and politic, and possesses a visitorial power over all the district societies; may increase their number or alter their geographical boundaries; and prescribe uniform rules and regulations for their government.

We have read the proceedings of this respectable society, but as might be expected of its first meeting, we find nothing of sufficient *general* interest to transfer to a journal, the principal circulation of which is in other states. They relate chiefly to its own organization, and the duties, objects, and limits of the district societies.

Several subjects of importance were, however, broached by the society, and will undoubtedly receive the grave attention of that body, at its future sessions, which are to be held biennially. Among these we may mention vaccination, a uniform course of medical studies, and the substitution of one or more boards of censors or examiners, to meet annually, for the present system of district censors. Many other subjects, will, no doubt, present themselves for the consideration, of the society hereafter; and we cherish the hope, that its exertions will be skilfully and unremittingly directed to the elevation of the medical character of the state.

The officers of the society are—

Dr. John Cotton, *President.*
" John C. Dunlavy, *Vice President.*
" John E. Bush, *Recording Secretary.*
" Benjamin Dickson, *Corresponding Secretary.*
" Samuel Parsons, *Treasurer.*

